# Evaluation of a Modified Mindfulness-Based Stress Reduction Intervention for Adults with Cerebral Palsy and Anxiety and/or Emotion Regulation Difficulties—A Randomised Control Trial

**DOI:** 10.3390/jcm13010001

**Published:** 2023-12-19

**Authors:** Hayley Smithers-Sheedy, Emma Waight, Katherine L. Swinburn, Fiona Given, Kate Hooke, Annabel Webb, Sarah McIntyre, Georgina Henry, Ingrid Honan

**Affiliations:** 1 Cerebral Palsy Alliance Research Institute, Sydney Medical School, The University of Sydney, Camperdown, NSW 2050, Australia; emma.waight@cerebralpalsy.org.au (E.W.); kath.swinburn@cerebralpalsy.org.au (K.L.S.); fgiven@optusnet.com.au (F.G.); kate.hooke@cerebralpalsy.org.au (K.H.); annabel.webb@cerebralpalsy.org.au (A.W.); smcintyre@cerebralpalsy.org.au (S.M.); georgina.henry@cerebralpalsy.org.au (G.H.); ingrid.honan@cerebralpalsy.org.au (I.H.); 2 UTS Disability Research Network, University of Technology, Ultimo, NSW 2007, Australia

**Keywords:** cerebral palsy, mindfulness-based stress reduction, mindfulness, anxiety, emotion regulation

## Abstract

Mindfulness-Based Stress Reduction (MBSR) has not yet been evaluated for people with cerebral palsy (CP). The aims of this randomised control trial were to investigate whether a modified telehealth MBSR program could improve mindfulness and reduce depression, anxiety, and emotion regulation difficulties among adults with CP with elevated anxiety and/or emotional regulation difficulties. Participants (*n* = 31) with elevated anxiety and/or emotion regulation difficulties and no/mild intellectual impairment were randomised to a modified telehealth MBSR program (90 min weekly, 9 weeks) group or a wait-list group. Measurements were collected prior to (T1), after (T2), and 8 weeks post-intervention (T3). The primary outcome was the mean between-group difference in the change in Cognitive and Affective Mindfulness Scale-R (CAMS-R) scores in T1–T2. The secondary outcomes included mean within-group differences over time for the CAMS-R total scores, Depression Anxiety and Stress Scale—21 subscales, and Difficulties in Emotion Regulation Scale (DERS) total t-score. We found no statistically significant between-group difference in mean change in mindfulness scores for T1–T2 (primary outcome). Secondary outcomes: The MBSR intervention group had improved CAMS-R scores with respect to T1–T2 and T1–T3; improved mean scores for Depression and Stress subscales for T1–T2; and improved DERS *t*-scores for T1–T2 and T1–T3. In conclusion, this study found no significant between-group difference for the primary outcome of mindfulness. The MBSR program was successfully modified for adults with CP and was effective in improving depression, stress, and emotion regulation. ACTRN12621000960853

## 1. Introduction

Cerebral palsy (CP) is a lifelong condition characterised by abnormal muscle tone and posture [1]. People affected by CP often have other associated impairments including intellectual, vision, communication, and hearing impairments and epilepsy [1]. A growing body of research is showing that people with CP also experience mood disorders at a higher rate than the general population [2,3,4]. In a recent survey of *n* = 42 adults with CP, we found that one third of the respondents had moderate to extremely severe depression scores, and 60% also had anxiety scores falling within this range [5,6]. These proportions are high when compared with population estimates reporting that around 10% of Australian adults suffer from depression and 13% are afflicted with anxiety disorder [7]. In this survey study, and in studies conducted on other diagnostic groups, including individuals with traumatic brain injuries, a positive association between symptoms of depression and anxiety and emotion regulation difficulties was identified [6,8]. Programs that are effective in improving emotion regulation such as Mindfulness-Based Stress Reduction (MBSR) may therefore have a role to play in improving mood disorders such as anxiety and depression [9,10].

Mindfulness consists of cultivating awareness of mind and body. It focuses on the non-judgmental awareness and acceptance of present-moment experiences [10]. Mindfulness-Based Stress Reduction (MBSR) is an eight-week evidence-based program developed by Professor Jon Kabat-Zinn in 1979. Although initially developed for stress management, in more recent years, MBSR has emerged as a psychological treatment used to treat psychiatric, psychosomatic, and physical conditions such as anxiety, stress, and pain [9,11]. Whilst the use of mindfulness has been explored in a wide variety of diagnostic groups [12,13,14], there are very few studies that have investigated the use of mindfulness to treat individuals with CP. The first study evaluated the effectiveness of a mindfulness-based movement programme (MiYoga) in improving attention among children with CP (*n* = 42, GMFCS I-III). This wait-list control trial showed significantly improved attention levels among members of the intervention group compared with the waitlist comparison group [15]. However, a follow-up study including the same group of participants found that the retention and long-term effects of MiYoga 6 months post-intervention were inconclusive, suggesting that booster sessions or strategies for integrating the program into daily routines may be required [16]. In a pilot study of *n* = 6 adults with CP and reported pain, Hoye et al. explored the potential benefits of a modified Mindfulness-Based Stress Reduction (MBSR) program using video conferencing [17]. The authors found that delivering the MBSR program via video-conferencing was feasible and that, on completion, participant pain catastrophizing and negative affect had significantly decreased [17]. The findings from this pilot study were promising and suggest that MBSR could be of benefit to adults with CP. However, as the sample size was small and there was no control group, it was not possible to generalise the findings beyond the pilot study.

In collaboration with people with lived experience of CP, it was determined that a further evaluation and trial of a modified MBSR program for adults with CP was warranted. The aims of this randomised control trial were to investigate whether a modified telehealth MBSR program could improve mindfulness and reduce depression, anxiety, and emotion regulation difficulties of adults with CP with elevated anxiety and/or emotional regulation difficulties. 

## 2. Materials and Methods

### 2.1. Study Design and Participants

This randomised control trial was conducted between May 2021 and December 2022. Participants were adults with CP recruited across four states/territories of Australia: New South Wales, the Australian Capital Territory, Queensland, and Victoria. Participants were invited to participate in the study through an invitation sent by email/mail through the CP registers based in each region. CP registers are confidential research databases, and one of their important functions is to support recruitment into ethically approved studies.

The eligibility criteria for participation were as follows:

- Adults with CP;
- Individuals 20–40 years of age;
- Individuals with the ability to speak conversational English, which could include the use of augmentative and alternative communication;
- Individuals self-identifying as having elevated anxiety or emotional regulation difficulties and one or more of the following scores on baseline assessment;
- DASS Anxiety raw score of four or higher;
- DERS total raw score of 99 or higher;
- Individuals that had not participated in active mindfulness training in the past year;
- Individuals with no reported moderate to severe intellectual impairment;
- Individuals identified as having functional hearing and vision;
- Individuals with access to the internet, a laptop, or a personal computer with a web camera and microphone for access to Microsoft Teams;
- Individuals committed to attending the 9-week course via telehealth on a Tuesday or Wednesday evening and to completing evaluations.

### 2.2. Randomisation and Blinding 

**Figure 1:**
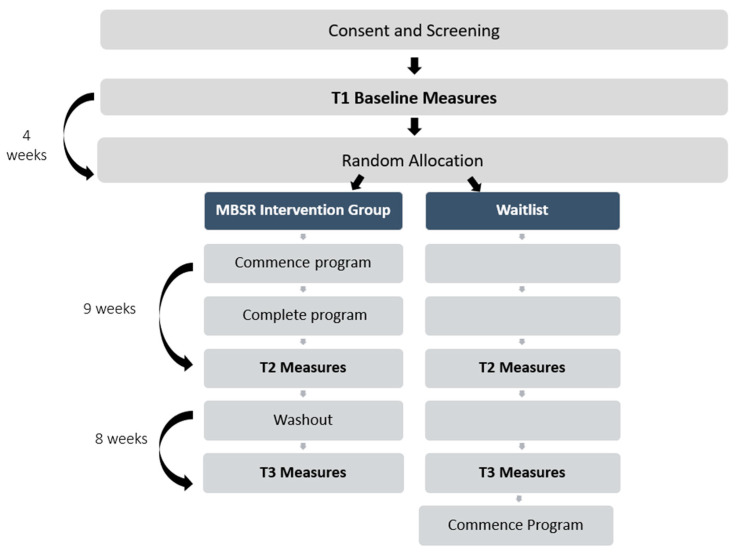
Study timeline.

Participants were randomised into treatment or wait list control groups (see Figure 1). Randomisation was stratified by Gross Motor Function Classification (GMFCS) I–II or GMFCS III–V using block randomisation using the randomisation function available in REDcap. Due to the study’s design, no blinding/concealment was possible.

### 2.3. Intervention 

The intervention was a 9-session modified MBSR program with weekly 90 min group telehealth sessions provided through Microsoft Office Teams (teleconferencing software, Microsoft Teams classic 1.6.00.34637). The modified MBSR program was presented and facilitated by a social worker (KH) who had completed facilitator training in MBSR and had extensive experience and expertise working with people with CP and in MBSR and facilitating groups. 

The modified program included the key components of available MBSR content drawn from published MBSR programs [18,19,20]. This content was modified to ensure that the session and home practice activities were accessible for people with CP. This involved modifying or removing standard activities or content that relied on gross/fine motor function, speech, or eating/drinking skills. Details of the modified MBSR program manual developed for this study are available on request from the corresponding author. 

Weekly topics were as follows:

1. Course overview and what is mindfulness?
2. The mind–body connection—how our body responds physiologically to anxiety and stress.
3. How to practice mindfulness meditation.
4. How mindfulness works with respect to anxiety and stress.
5. Mindfulness for the body.
6. Deepening your practice—sitting meditation.
7. Meditation for anxiety and stress.
8. Loving kindness meditation.
9. Interpersonal mindfulness and how to make mindfulness part of your everyday life.

Participants were provided with a manual that included information about the targeted constructs, a list of activities for home practice, exercise sheets to record their experiences, and audio file links of the mindfulness meditation activities completed in the sessions. Participants who missed a session were also able to access a recording of the missed session in the following week. 

The course facilitator met with the research team after each week to discuss any issues in delivering the course content via telehealth. 

### 2.4. Outcome Measures 

Measures, all of which were self-reported, were collected prior to (T1), after (T2), and 8 weeks post-intervention (T3) using a secure online survey conducted using REDCap (Figure 1, Table 1 and Table 2). The course facilitator was not involved in data collection.

The primary outcome measure was the mean between-group difference in the change in Cognitive and Affective Mindfulness Scale-R (CAMS-R) [21] total scores from T1 to T2. The CAMS-R is a self-report measure for mindfulness behaviour/experiences. A primary endpoint of the period immediately post-intervention was selected to gauge differences in mindfulness knowledge, practice, and behaviours, the primary aim of the MBSR intervention.

Secondary outcomes were as follows:

- The mean between-group difference (MBSR vs Control) in CAMS-R scores from T1 to T3.
- The mean within-group difference in the CAMS-R total score from T1 to T2 and T1 to T3 for the MBSR intervention group.
- The mean within-group difference from T1 to T2 and T1 to T3 with regard to the following:
  - Depression Anxiety and Stress Scale—21 (DASS-21);
  - Depression;
  - Anxiety;
  - Stress;
  - Difficulties in Emotion Regulation Scale (DERS) Total Raw Score.
- The mean within-group differences from T1 to T2 and T1 to T3 with regard to the following:
  - Brief Pain Inventory (BPI) short-form pain intensity and interference scores;
  - Point changes in ratings on a 10-point rating scale between baseline and follow-up time points across 5 individual specific anxiety-inducing situations.

### 2.5. Statistical Analysis 

We aimed to detect a large effect size equivalent to Cohen’s d of 0.8 in the primary outcome, with 80% power and a two-sided significance level of alpha = 0.05 assuming a standard deviation of SD = 3.05 (as suggested by Lau et al. (2015)) [32]. To achieve the desired statistical power using a baseline-adjusted ANCOVA model and assuming a moderately high within-subject correlation of r = 0.7 between baseline and post-intervention scores on the primary outcome, a minimum sample size of 13 participants in both the MBSR intervention and control group (total *n* = 26) was required. Accounting for a dropout rate of 10%, a minimum of *n* = 30 (15 in each group) participants were required. 

Descriptive statistics were used to examine the survey responses at each time point. Following this, normality of continuous variables was assessed using the Shapiro–Wilk test, visual inspection of the histogram, and a box plot for outliers. Two-sample *t*-tests were employed to compare baseline measurements of demographic and clinical variables between groups. Analysis of covariance (ANCOVA) was then used to compare the changes in CAMS-R between the MBSR intervention and control groups. Preliminary imputation of missing data for the primary and secondary outcomes at T2 and T3 was carried out using the last-value-carried-forward method. Both observed scores (i.e., a complete case analysis) and last-value-carried-forward scores were calculated and reported when examining the change in mean CAMS-R scores over time. Paired *t*-tests were conducted by using the last-value-carried-forward method to measure change in scores within groups for the secondary outcomes (Anxiety, Stress, and Depression scores from the DASS-21; DERS total scores; pain intensity and interference scores; and point changes in ratings on a 10-point rating scale across 5 individual-specific anxiety-inducing situations). *p*-values of <0.05 were considered statistically significant. Effect sizes and 95% confidence intervals were computed. All analyses were conducted in accordance with the intention-to-treat principle to preserve randomisation.

Analysis was completed using R version 4.3.1.

## 3. Results 

**Figure 2:**
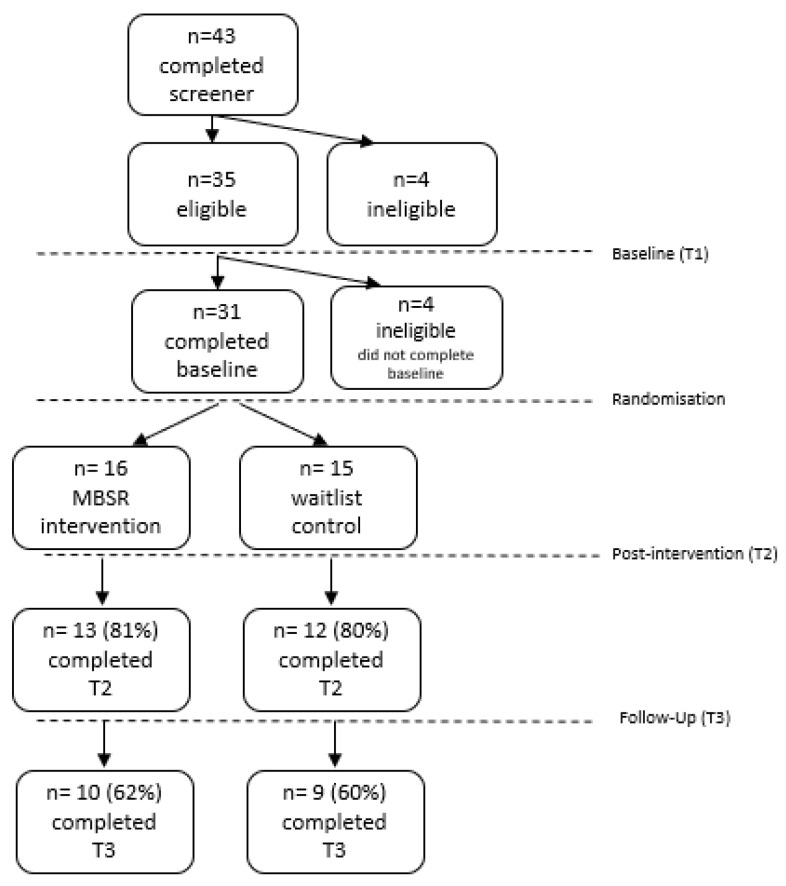
Participant flow chart.

Of the *n* = 35 adults who satisfied the inclusion criteria during screening, *n* = 31 continued to complete the baseline questionnaires and were recruited and randomised (16 of whom were randomised to the MBSR intervention, and the other 15 were randomised to the control group; Figure 2). Four eligible people did not complete the baseline measures and so did not continue to randomisation. The completion rate of follow-up measures at T2 and T3 was around 60% (see Figure 2). 

There were no significant differences between the intervention and control groups at baseline in terms of socio-demographic (Table 3) or clinical characteristics (except pain intensity) (Table 4). Just under half of all the participants had one or more self-reported psychological disorders, including anxiety disorder/generalised anxiety disorder (*n* = 9), depression (*n* = 5), post-traumatic stress disorder (PTSD) and complex post-traumatic stress disorder C-PTSD) (*n* = 4), and other (*n* = 6), which included bipolar disorder, personality disorders, gender dysphoria, and developmental psychological conditions. Amongst people with a self-reported psychological disorder, the average age of diagnosis was 14.5 years (SD 5.41).

### 3.1. Dose

On average, the participants attended seven of the nine sessions. At T2, the participants in the MBSR group reported they spent, on average, ≈3 h per week (range 1–4 h) practicing mindfulness-based skills outside of the program. At T3, this dropped to 1 1/2 h per week (range of 0–3 h). 

### 3.2. Modifications to Course Content during the Study Period 

There were no amendments made to the modified MBSR telehealth program during the evaluation. 

### 3.3. Primary Outcome Measure

**Figure 3:**
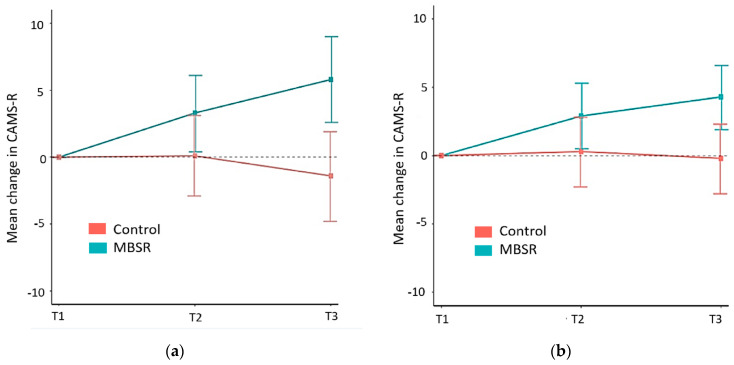
(**a**) Raw mean change in Cognitive and Affective Mindfulness Scale-R (CAMS-R) scores at baseline (T1), post-intervention (T2), and at follow-up (T3); (**b**) mean change in CAMS-R scores using last-value-carried-forward method at baseline (T1), post-intervention (T2), and at follow-up (T3).

The primary outcome was the mean group difference in change in CAMS-R total scores (mindfulness measure) between the baseline (T1) and post-intervention (T2) timepoints. This was calculated using both observed scores in a complete-case analysis and last-value-carried-forward imputation to account for missing data (Figure 3a,b). Using the last-value-carried-forward method, it was determined that the mean group difference in change of mindfulness (CAMS-R scores) between the MBSR and control groups was not statistically significant (1.8 (−2.1, 5.6), *p* = 0.344) (Figure 3b). 

An analysis of missing primary outcome data revealed that the participants with better emotion regulation scores, lower depression scores, higher levels of social support, and who did not have motor speech impairments at baseline were less likely to complete the T2 and T3 surveys. 

### 3.4. Secondary Outcome Measures

Analysis of mindfulness (CAMS-R scores) between the baseline (T1) and 8-week follow-up (T3) time points revealed a significant between-group difference in CAMS-R mean raw scores in the complete-case analysis (Figure 3a). However, when we recalculated this to account for missing data using the last-value-carried-forward imputation method, this difference did not reach statistical significance (3.7 points (−0.1, 7.5), *p* = 0.157) (Figure 3b). 

**Figure 4:**
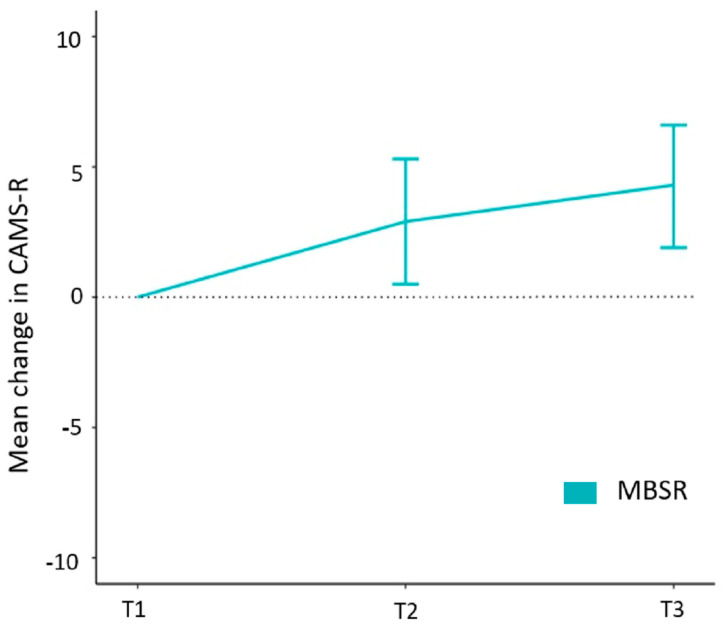
MBSR intervention group: within-group mean change in Cognitive and Affective Mindfulness Scale-R (CAMS-R) scores using last-value-carried-forward method at baseline (T1), post-intervention (T2), and at follow-up (T3).

All remaining secondary outcome measures pertain to evaluation of within-group changes for the MBSR intervention group. Here, analyses revealed an increase in mindfulness (CAMS-R scores) by an average of 2.9 (0.5, 5.3) points between T1 and T2 (*p* = 0.019). Significant increases in mean mindfulness (CAMS-R scores) were also observed between T1 and T3, rising by an average of 4.3 (1.9, 6.6) points (*p* = 0.008), as determined using the last-value-carried-forward method (Figure 4 and Supplemental Table S1).

An analysis of the within-group changes for the MBSR intervention group also revealed a significant mean decline in DASS-21 scores post-intervention (T2) for the Depression [−2.7 (−4.7, −0.7), *p* = 0.013] and Stress [−2.1(−3.7, −0.4), *p* = 0.023] subscales. However, the observed improvements in scores for these subscales did not remain statistically significant at the 8-week follow-up (T3) (Depression: *p* = 0.132; Stress: *p* = 0.420) (Supplemental Table S1). 

Analysis of within-group changes for the MBSR intervention group for emotion regulation (DERS) revealed a mean decline in Total DERS *t*-scores of −6.6 (−11.1, −2.2) at T2 (*p* = 0.037) and by −7.7 (−12.1, −3.3) at T3 (*p* = 0.027), demonstrating significant improvements in emotion regulation. 

There were no significant within-group differences identified between the post-intervention or follow-up timepoints for the BPI interference or intensity scores in the MBSR group (Supplemental Table S1). 

The anxiety-inducing situations that were provided by participants varied widely in terms of the number of situations entered (between zero and five) and the frequency of these situations (e.g., a family member has COVID-19, I am applying for a job, my routine is changing, etc.). As there was such a great deal of variation and a considerable number of missing data at the post-intervention and follow-up timepoints, it was determined that an analysis of the available data would not be informative. 

At baseline, 40% (*n* = 12) of all the participants indicated that the COVID-19 pandemic had impacted their wellbeing in the last week. This proportion only changed marginally over time. There was no significant difference between the MBSR and control groups in terms of the proportion reporting that COVID-19 had impacted their well-being at T1 (*p* = 0.46), T2 (*p* = 0.56), or T3 (*p* = 0.99). 

## 4. Discussion

This study is the first randomised control study to evaluate whether a modified MBSR program could improve mindfulness and reduce depression, anxiety, and emotion regulation difficulties among adults with CP and elevated anxiety and/or emotional regulation difficulties. Looking first at mindfulness (primary outcome), we found no significant between-group differences in the primary outcome measure (CAMS-R scores). However, analysis of the secondary outcome data revealed within-group improvements in mindfulness for the MBSR intervention group immediately after and 8 weeks post-intervention (*p* < 0.05), demonstrating the usefulness of this modified telehealth MBSR intervention. 

The modifications to published MBSR programs used in this study primarily included the modification or removal of motor-based activities, for example, mindful eating, walking meditation, speaking tasks, and tasks with a focus on the mobilisation of different body parts. These changes were made to ensure the program was accessible to adults with CP regardless of their motor type, level of functional motor limitation, or communication method. In a companion qualitative study, we invited the participants to participate in focus groups to provide more in-depth feedback about the content and their experience of the MBSR program. Importantly, this companion study, which will be published soon, identified both additional refinements that could be made to the program and participant reflections of this program that fell outside of the primary and secondary outcome measures reported here. 

Significant improvements in within-group MBSR intervention group scores were observed for depression, stress, and emotion regulation immediately after the intervention, with improvements in emotion regulation maintained at 8 weeks (*p* < 0.05). Emotion regulation is important. Being able to attend to, experience, and regulate our emotions helps us to feel in control and to respond in a socially acceptable manner to both the pressures and joys of life, which are key aspects for both our individual well-being and our relationships [33,34,35]. In line with previous research conducted across a range of populations, this mindfulness intervention was effective in improving emotion regulation, which was sustained at 8 weeks [36,37,38,39]. Considering the well-recognised relationship between emotion regulation and mood [40], it was thus unsurprising that improvements in emotion regulation were mirrored by improvements in depression scores post-intervention. However, as emotion regulation and anxiety are so closely related, we would have expected a more substantial decline in anxiety scores [41]. There are a number of possibilities that may explain this. One possibility to consider is that whilst MBSR has been shown to be effective for generalised anxiety disorders [10], a number of participants in this study had more complex anxiety disorders, e.g., anxiety plus PTSD. Another factor that may have impacted the level of anxiety of participants was the COVID-19 pandemic and the sporadic periods of lockdown that were occurring across New South Wales and Victoria during the intervention period. Levels of anxiety were higher during the pandemic in the general population, with people suffering from pre-existing mental health issues at increased risk [42]. 

The strengths of this study include its design; i.e., it was a randomised control trial. Additionally, the clinical characteristics of the participants were largely representative of the general CP population, excluding intellectual impairment [43]. The participants had a range of CP motor types, Gross Motor Function Classification System levels, and degrees of communication function. Another strength of this study was the use of telehealth to conduct this intervention. Australia is a geographically large country, and by providing this program as a telehealth intervention, many service access barriers were removed (e.g., travel time and associated costs). Providing this modified MBSR program via telehealth was also relatively inexpensive and required only one course facilitator. 

A limitation of this study was that adults with CP and intellectual impairment were not eligible to participate. This limits the generalisability of our findings to adults with CP who do not have an intellectual impairment. As a result, future studies will be needed to assess the effectiveness of a modified MBSR program for adults with CP and intellectual impairment. Some additional program modifications for this group that could be considered based on our clinical experience and the literature include shorter sessions, conducted over more weeks, and sessions provided in-person with a buddy to support additional practice [44,45]. The main limitation of this study was the loss of participants to follow-up. To account for this, we analysed the data using the last-value-carried-forward method to report these findings as conservatively as possible and to protect against bias from potentially non-random dropout. Using last-value-carried-forward analysis, much smaller effect sizes of *d* = 0.3 at T2 and *d* = 0.6 at T3 were obtained, and hence the statistical power of the study for detecting a between-group difference was reduced. Future studies may wish to consider increasing the sample size to provide sufficient power for larger loss-to-follow-up rates or more conservative analyses such as last-value-carried-forward approaches. 

## 5. Conclusions

Mood disorders and emotion regulation difficulties are over-represented among adults with CP. MBSR programs such as the modified version trialled in this study are emerging as important tools for supporting better mental health. Future studies examining the impact of MBSR on mindfulness in this population should consider larger sample sizes to better account for loss to follow-up and to ensure sufficient power. Despite this limitation, this study shows that MBSR programs can be modified to be accessible for adults with CP and are effective in improving depression, stress, and emotion regulation.

## Figures and Tables

**Table 1:** Measures.

Variables	Measure	Description
**Mindfulness behaviour and experiences**	**The Cognitive and Affective Mindfulness Scale-R (CAMS-R) [** **21** **]**	A 12-item measure designed to capture a broad conceptualisation of mindfulness with language that is not specific to any particular type of meditation training. Respondents self-report their responses to the 12 items using a 4-point Likert scale (ranging from rarely/not at all to almost always). Items requiring reverse scoring are coded, and item scores are summed to obtain a total score, with higher CAMS-R scores indicating adequate sensitivity to self-reported changes [22].
**Depression, anxiety, and stress**	**Depression Anxiety and Stress Scale—21 (DASS-21) [** **5** **]**	DASS-21 is a valid and reliable categorical conception of depression, anxiety, and stress dimensions. DASS-21 scores indicate the degree to which someone is experiencing symptoms, but it is not a diagnostic tool. It has been used across a wide range of populations, including adolescents and adults with developmental disabilities (e.g., Down’s Syndrome).
**Emotion** **regulation**	**Difficulties in** **Emotion Regulation Scale (DERS) [****23**,**24****]**	Widely used self-report measure of subjective emotion regulation ability. It has been used previously by the investigators in a study on adults with CP [6].
**Pain**	**Brief Pain Inventory (BPI) pain intensity and interference scores [****25**,**26****]**	The BPI is widely used to assess the severity and impact of pain on daily functions. It is a recommended outcome measure for clinical trials assessing chronic pain as per the IMMPACT guidelines (2005) [25,26].
**Anxiety**	**Changes in ratings of 5 specific anxiety-inducing situations**	Participants were asked to individually identify 5 anxiety-inducing situations and rate the level of anxiety induced by each situation on a 10-point rating scale.
**Co-variates**	**Measure**	Description
**Socio-demographic and clinical characteristics**	**Survey**	Self-report survey with a range of variables, including age, gender, employment, and accommodation. Participants were also asked to indicate their motor type and topography of CP (spastic hemiplegia/monoplegia, spastic diplegia, spastic tri/quadriplegia, ataxic, and/or dyskinetic). Gross motor limitations were classified using the Gross Motor Function Classification System (GMFCS) [27]. Speech and communication function were classified using the Communication Function Classification System [28] and Viking Speech Scale [29] and via the use of Augmentative and Alternative Communication.
**Perceived** **social support**	**The Multidimensional Scale of Perceived Social Support [** **30** **]**	A 12-item self-report measure of perceived adequacy of social support from three sources: family, friends, and significant other. A total score on this measure ranging from 61–84 is indicative of high perceived support.
**Sense of community**	**Sense of Community Index-2 [** **31** **]**	A 24-item self-report measure with responses recorded from a four-point Likert scale with scores ranging from 0 to 3 for a maximum score of 72 and a maximum subscale score of 18. Higher scores in this index suggest a greater sense of community.
**COVID-19-related well-being**	**Single survey question**	A single survey question was included to ascertain whether COVID-19 had impacted participant well-being in the last week.

**Table 2:** Data collection and timepoints.

Assessment/Procedure	Screening	Baseline T1	Post MBSR Intervention T2	Follow-Up T3
Screening re-inclusion/exclusion criteria	✓			
**Covariates**				
Socio-demographic and clinical characteristics		✓		
The Multidimensional Scale of Perceived Social Support		✓		
Sense of Community Index-2		✓		
COVID-19-related well-being		✓	✓	✓
**Outcomes**				
Cognitive and Affective Mindfulness Scale-R		✓	✓	✓
Depression, Anxiety, and Stress Scale—21		✓	✓	✓
Difficulties in Emotion Regulation Scale		✓	✓	✓
BPI Pain interference score		✓	✓	✓
BPI Pain intensity score		✓	✓	✓
Scores regarding 5 anxiety-inducing situations		✓	✓	✓
Practice of mindfulness-based skills			✓	✓

**Table 3:** Socio-demographic characteristics at baseline.

	Total *n* = 31	MBSR Intervention *n* = 16	Control *n* = 15	*p-*Value
**Age, years, mean (SD)**	25.3 (3.9)	26.2 (4.3)	24.2 (3.2)	0.10
Years, *n* (%)				
20–24	13 (41.9%)	4 (25.0%)	9 (60.0%)	0.20
25–29	14 (45.2%)	9 (56.2%)	5 (33.3%)	
30–35	4 (12.9%)	3 (18.8%)	1 (6.7%)	
**Gender, *n* (%)**				
Male	8 (25.8%)	5 (31.2%)	3 (20.0%)	0.40
Female	21 (67.7%)	11 (68.8%)	10 (66.7%)	
Non-binary	2 (6.5%)	0 (0.0%)	2 (13.3%)	
**Employment, *n* (%)**				
Paid employment	12 (38.7%)	9 (56.3%)	3 (20.0%)	0.14
Student	9 (29.0%)	3 (18.8%)	6 (40.0%)	
Volunteer/not engaged in paid employment	10 (32.3%)	4 (25.0%)	6 (40.0%)	
**Living situation, *n* (%)**				
With parents/family member	21 (67.7%)	11 (68.8%)	10 (66.7%)	0.49
Alone/with spouse	6 (19.4%)	4 (25.0%)	2 (13.3%)	
Share house/supported accommodation	4 (12.9%)	1 (6.2%)	3 (20.0%)	
**Children, *n* (%)**				
Yes	0			
No	31 (100.0%)			
**Receive care support for daily living activities, *n* (%)**				
Yes	20 (64.5%)	11 (68.8%)	9 (60.0%)	0.70
No	11 (35.5%)	5 (31.2%)	6 (40.0%)	0
**Support person, *n* (%)**				
Partner/spouse	3 (9.7%)	2 (12.5%)	1 (6.7%)	0.99
Parent	22 (71.0%)	11 (68.8%)	11 (73.3%)	
Friend	1 (3.2%)	1 (6.3%		
Sibling	1 (3.2%)	0	1 (6.7%)	
Case worker/advocate	1 (3.2%)	0	1 (6.7%)	
Other	3 (9.7%)	2 (12.5%)	1 (6.7%)	
**The Multidimensional Scale of Perceived Social Support, mean (SD)**				
Total	60.4 (17.1)	56.6 (16.3)	64.4 (17.6)	0.20
Friends	18.5 (6.4)	16.6 (6.8)	20.7 (5.3)	0.07
Family	21.2 (6.7)	20.9 (6.2)	21.6 (7.4)	0.80
Significant Other	20.6 (7.0)	19.1 (6.6)	22.1 (7.2)	0.20
**Sense of Community Index-2, mean (SD)**				
Total	65.1 (19.8)	65.1 (18.6)	65.1 (21.6)	0.99
Reinforcement of needs	16.4 (4.8)	16.4 (4.2)	16.5 (5.5)	0.96
Membership	15.7 (5.3)	15.6 (5.2)	15.9 (5.6)	0.88
Influence	15.5 (5.1)	15.6 (5.1)	15.3 (5.2)	0.90
Shared emotional connection	17.5 (5.4)	17.6 (5.0)	17.4 (5.9)	0.91

**Table 4:** Clinical characteristics at baseline.

	Total *n* = 31	Intervention *n* = 16	Control *n* = 15	*p*-Value
**Gross Motor Function (GMFCS), *n* (%)**				
Level I–III	24 (77.4%)	12 (75.0%)	12 (80.0%)	0.99
Level IV–V	7 (22.6%)	4 (25.0%)	3 (20.0%)	
**CP Motor Type, *n* (%)**	12 (38.7%)	5 (31.2%)	7 (46.7%)	0.79
Spastic hemiplegia/monoplegia				
Spastic diplegia	9 (29.0%)	6 (37.5%)	3 (20.0%)	
Spastic triplegia or quadriplegia	6 (19.4%)	3 (18.8%)	3 (20.0%)	
Other (Ataxia, mixed type, not stated)	4 (12.9%)	2 (12.5%)	2 (13.3%)	
**Communication Function (CFCS), *n* (%)**				
Level I	22 (71.0%)	12 (75.0%)	10 (66.7%)	0.70
Level II–V	9 (29.0%)	4 (25.0%)	5 (33.3%)	
**Viking Speech Scale (VSS)** [29]**, *n* (%)**				
Level I	20 (64.5%)	11 (68.8%)	9 (60.0%)	0.70
Level II–IV	11 (35.5%)	5 (31.2%)	6 (40.0%)	
**Augmentative/alternative communication system used, *n* (%)**				
Yes	4 (12.9%)	2 (12.5%)	2 (13.3%)	0.99
No	27 (87.1%)	14 (87.5%)	13 (86.7%)	
**Cognitive and Affective Mindfulness Scale-R, mean (SD)**	27.9 (6.3)	26.7 (6.2)	29.3 (6.3)	0.30
**Depression, Anxiety and Stress Scale-21, mean (SD)**				
Total	22.7 (10.3)	22.7 (10.9)	22.8 (10.0)	0.99
Depression	7.1 (4.9)	7.3 (4.5)	6.9 (5.5)	0.80
Anxiety	6.2 (3.7)	6.4 (4.4)	6.1 (3.0)	0.80
Stress	9.5 (3.7)	9.1 (3.0)	9.9 (4.6)	0.60
**Difficulties in Emotion Regulation Scale, mean (SD)**				
Total	59.6 (14.9)	60.4 (14.4)	58.4 (16.2)	0.70
Non-acceptance	60.7 (10.1)	62.7 (8.1)	58.1 (12.1)	0.20
Goals	59.4 (11.4)	61.7 (9.3)	56.2 (13.6)	0.20
Impulse	56.2 (14.9)	56.9 (14.6)	55.3 (16.0)	0.80
Awareness	55.4 (14.7)	54.1 (12.2)	57.2 (18.0)	0.60
Strategy	56.4 (13.4)	58.8 (14.2)	53.3 (12.0)	0.30
Clarity	56.1 (13.3)	55.7 (13.1)	56.7 (14.0)	0.80
**Brief Pain Inventory, *n* (%)**				
Interference	18.8 (19.5)	17.9 (17.6)	19.7 (21.9)	0.80
Intensity	4.0 (2.0)	3.1 (2.0)	4.9 (1.5)	0.01
**Situation Anxiety Ratings, mean (SD)**	6.7 (1.6)	6.3 (1.8)	7.2 (1.4)	0.10
**COVID impacted well-being in the last week, *n* (%)**				
No	18 (60.0%)	11 (68.8%)	7 (50.0%)	0.46
Yes	12 (40.0%)	5 (31.2%)	7 (50.0%)	

## Data Availability

Please contact the corresponding author to request access to aggregated data.

## Supplementary Materials

The following supporting information can be downloaded at https://www.mdpi.com/article/10.3390/jcm13010001/s1. Table S1: Mean within-group changes from baseline measurement, post-intervention (T2), and follow-up (T3) determined via last-value-carried-forward analysis.
