# Supplementary material for: Evaluation of a Modified Mindfulness-Based Stress Reduction Intervention for Adults with Cerebral Palsy and Anxiety and/or Emotion Regulation Difficulties—A Randomised Control Trial
Source: J Clin Med. 2023 Dec 19;13(1):1. doi: 10.3390/jcm13010001 (PMC10779681; doi:10.3390/jcm13010001)
Supplement: Supplementary file 1 [file jcm-13-00001-s001.zip › jcm-2727242-supplementary.pdf]

Supplementary Materials:

Supplemental Table S1. Mean within-group changes from baseline measurement, post-intervention (T2), and follow-up (T3) determined via last-value-carried-forward analysis.

	**Post-intervention (T2)**		**Follow-up (T3)**	
	**MBSR Intervention Group**	Control Group	MBSR Intervention Group	Control Group
**CAMS-R, mean change (95% CI)**	**2.9 (0.5, 5.3)**	0.3 (-2.3, 2.8)	**4.3 (1.9, 6.6)**	-0.2 (-2.8, 2.3)
**DASS-21, mean change (95% CI)**				
Total	**-5.6 (-9.8, -1.5)**	-1.8 (-6.3, 2.7)	-3.9 (-8.1, 0.3)	0.4 (-4.1, 4.8)
Depression	**-2.7 (-4.7, -0.7)**	-1.4 (-3.5, 0.8)	-1.8 (-3.8, 0.2)	-0.9 (-3.0, 1.3)
Anxiety	-1.0 (-2.4, 0.4)	0.0 (-1.5, 1.5)	-1.2 (-2.6, 0.2)	0.4 (-1.1, 2.0)
Stress	**-2.1 (-3.7, 0.4)**	-0.6 (-2.4, 1.1)	-1.0 (-3.7, 0.6)	-0.6 (-2.4, 1.1)
**DERS T-score, mean change (95% CI)**				
Total	**-6.6 (-11.1, -2.2)**	-3.5 (-8.7, 1.6)	**-7.7 (-12.1, -3.3)**	-2.7 (-7.9, 2.4)
Non-acceptance	**-3.3 (-6.5, -0.1)**	-2.1 (-5.8, 1.6)	**-3.8 (-7.0, -0.6)**	-0.7 (-3.0, 4.4)
Goals	**-3.7 (-7.3, -0.1)**	-2.4 (-6.5, 1.8)	**-4.3 (-7.9, -0.7)**	0.8 (-3.4, 4.9)
Impulse	**-3.4 (-8.6, 1.8)**	-4.0 (-10.0, 2.0)	**-5.6 (-10.8, -0.4)**	-0.2 (-6.2, 5.80)
Awareness	-4.0 (-8.4, -0.4)	-0.8 (-5.9, 4.2)	**-4.8 (-9.2, -0.4)**	-1.9 (-6.9, 3.2)
Strategy	**-7.6 (-11.7, -3.5)**	-0.3 (-5.0, 4.5)	**-7.4 (-11.5, -3.3)**	-1.2 (-6.0, 3.6)
Clarity	**-4.2 (-8.0, -0.4)**	-2.0 (-6.4, 2.4)	**-5.8 (-9.6, -2.0)**	-2.9 (-7.3, 1.5)
**BPI mean change (95% CI)**				
Interference	-6.8 (-20.7, 7.1)	4.9 (-9.5, 19.2)	6.9 (-7.0, 20.8)	12.1 (-2.3, 26.4)
Intensity	0.8 (-0.2, 1.7)	-0.9 (-1.9, 0.2)	0.6 (-0.3, 1.6)	-0.9 (-2.0, 0.1)

Bold indicative of significant within group change from baseline measurement.
